# Patterns of evolution of host proteins involved in retroviral pathogenesis

**DOI:** 10.1186/1742-4690-3-11

**Published:** 2006-02-07

**Authors:** Millan Ortiz, Gabriela Bleiber, Raquel Martinez, Henrik Kaessmann, Amalio Telenti

**Affiliations:** 1 Institute of Microbiology and University Hospital, University of Lausanne, Switzerland; 2 Center for Integrative Genomics, University of Lausanne, Lausanne, Switzerland

## Abstract

**Background:**

Evolutionary analysis may serve as a useful approach to identify and characterize host defense and viral proteins involved in genetic conflicts. We analyzed patterns of coding sequence evolution of genes with known (*TRIM5*α and *APOBEC3G*) or suspected (*TRIM19*/*PML*) roles in virus restriction, or in viral pathogenesis (*PPIA*, encoding Cyclophilin A), in the same set of human and non-human primate species.

**Results and conclusion:**

This analysis revealed previously unidentified clusters of positively selected sites in *APOBEC3G *and *TRIM5*α that may delineate new virus-interaction domains. In contrast, our evolutionary analyses suggest that *PPIA *is not under diversifying selection in primates, consistent with the interaction of Cyclophilin A being limited to the HIV-1M/SIVcpz lineage. The strong sequence conservation of the *TRIM19/PML *sequences among primates suggests that this gene does not play a role in antiretroviral defense.

## Background

Evolutionary genomics approaches have been proposed as powerful tools to identify protein regions relevant for host-pathogen interactions [1]. Identifying signatures of genetic conflict can open the way to biological testing of hypotheses regarding the function of host proteins. In retrovirology, the utility of this approach was recently demonstrated in evolutionary analyses of the antiretroviral defense genes *TRIM5*α, encoding a retrovirus restriction factor targeting the viral capsid [2,3], and *APOBEC3G*, coding for a cytidine deaminase that hypermutates viral DNA in primates [4-6]. Both genes were shown to have been shaped by positive selection, which led to the rapid fixation of adaptive amino acid replacement substitutions. The two genes revealed two different patterns of positive selection: a localized region of rapid change in *TRIM5*α [3], and a pattern where positively selected residues are scattered throughout the sequence in *APOBEC3G *[5].

To assess the potential of an evolutionary approach to identify further primate genes/proteins involved in virus defense, we analyzed coding sequence evolution of two additional genes, *TRIM19 *(*PML*) and *PPIA*, and reassessed the selective signatures of *TRIM5*α and *APOBEC3G *in a common set of primates, representing 40 million years of evolution [7]. TRIM19 (PML) was proposed to possess anti(retro)viral activity [8,9], while Cyclophilin A, encoded by *PPIA *(peptidyl-prolyl cis-trans isomerase), is incorporated into HIV-1 particles through an interaction with the viral capsid [10]. Cyclophilin A is incorporated only into viral particles of viruses of the HIV-1M/SIV_CPZ _lineage, where it is required for viral replication [11].

**Figure 1:**
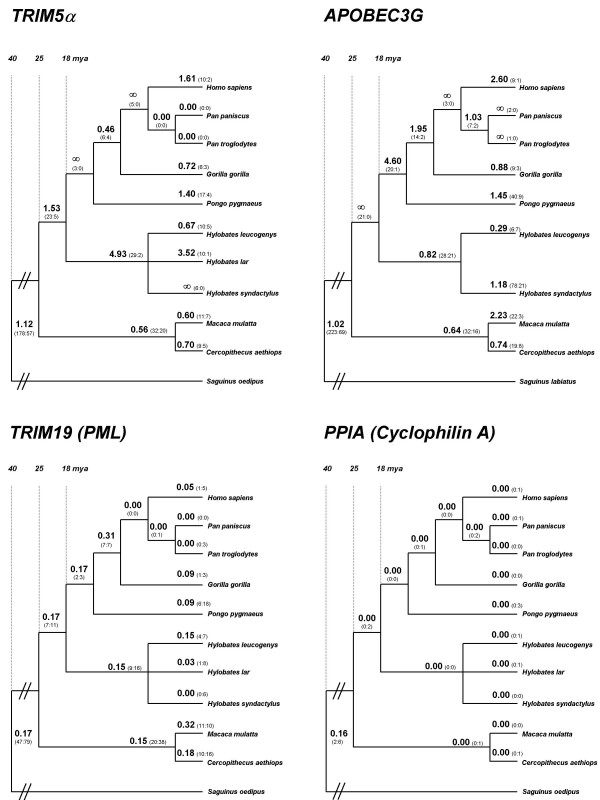
**Phylogenetic trees of candidate antiviral defense genes**. *K*_*A*_/*K*_*S *_values and the estimated number of nonsynonymous and synonymous substitutions (in parentheses) for each branch are indicated. Approximate divergence times in millions of years (mya) are shown [7].

To trace the evolutionary history of these genes, we first sequenced their coding regions from eleven primate species [see Additional files 1 and 2]. We then analyzed their substitutional patterns in the framework of the accepted primate phylogeny [7] using several codon-based maximum likelihood procedures as implemented in the codeml tool of the PAML program package [12] (Figure 1).

To obtain an overview of the coding sequence evolution, we estimated the number of nonsynonymous (*K*_A_) over synonymous (*K*_S_) substitutions per site (averaged over the entire sequence) for each branch of the trees using the free-ratio model of codeml [12]. Similarly to previous reports [3,5,6], this analysis revealed generally high *K*_A_/*K*_S _values on the different branches of the *TRIM5*α and *APOBEC3G *trees (average *K*_A_/*K*_S _~1.1 for both genes), indicating that these genes show accelerated amino acid replacement rates due to the action of positive selection [13]. In contrast, *PPIA *and *TRIM19 *(PML) show low *K*_A_/*K*_S _values (0.05 and 0.15, respectively, when averaged over the entire tree), suggesting that their protein sequences have been strongly preserved by purifying selection (Figure 1).

In more detailed analyses, we then utilized models that allow for different *K*_A_/*K*_S _rates at different sites of the sequences, because adaptive evolution often occurs at a limited number of sites [14]. We first compared a null model ("M1a", [15,16]), which assumes two site classes (sites under purifying selection and neutrally evolving sites), to an alternative model ("M2a", [15,16]), which adds a third site class that allows for sites with *K*_A_/*K*_S _> 1, using likelihood ratio tests [17]. This comparison revealed that the alternative model provides a significantly better fit (*P *< 10^-30^) for the *TRIM5*α and *APOBEC3G *genes than the null model, whereas the null model could not be rejected for *TRIM19 *and *PPIA *(Table 1). The *K*_A_/*K*_S _for the additional site class is larger than 1 for both *TRIM5*α (*K*_A_/*K*_S _~6.4) and *APOBEC3G *(*K*_A_/*K*_S _~4.4), strongly suggesting adaptive protein evolution driven by positive selection at a subset of sites. Thus, this analysis supports the hypothesis that *TRIM5*α and *APOBEC3G *evolved under positive selection. Contrary to this, nearly all sites of *TRIM19 *and *PPIA *(91.5% and 100%, respectively) are under purifying selection (Table 1).

**Table 1:** Codeml analyses using site-specific models.

***TRIM5*α**					
Site-specific Models^a^	ω_0_^b^	ω_1_^c^	ω_2_^d^	LogL	Sites with ω > 1 ^e^
C: M1a	0.00 (34.91%)	1.00 (65.09%)		-4117.12	
D: M2a	0.00 (26.04%)	1.00 (61.67%)	6.37* (12.29%)	-4087.97	11 sites

***APOBEC3G***					

Site-specific Models	ω_0_	ω_1_	ω_2_	LogL	Sites with ω > 1
C: M1a	0.03 (37.56%)	1.00 (62.44%)		-4187.55	
D: M2a	0.00 (28.28%)	1.00 (48.60%)	4.40* (23.11%)	-4148.85	24 sites

***TRIM19 (PML)***					

Site-specific Models	ω_0_	ω_1_	ω_2_	LogL	Sites with ω > 1
C: M1a	0.09 (91.47%)	1.00 (8.53%)		-5215.40	
D: M2a	0.11 (97.25%)	1.00 (0.00%)	2.5 (2.75%)	-5214.46	n/a ^f^

***PPIA (Cyclophilin A)***					

Site-specific Models	ω_0_	ω_1_	ω_2_	LogL	Sites with ω > 1
C: M1a	0.05 (100%)	1.00 (0%)		-751.04	
D: M2a	0.05 (100%)	1.00 (0.00%)	1.00 (0.00%)	-751.04	n/a ^f^

^a ^the likelihood models used are described in the text

^b ^class of sites under purifying selection

^c ^class of sites evolving neutrally

^d ^class of sites that may show *K*_A_/*K*_S _> 1

^e ^sites pinpointed to be under positive selection by Bayes Empirical Bayes analysis

^f ^test not applicable (M1a and M2a not significantly different)

**Figure 2:**
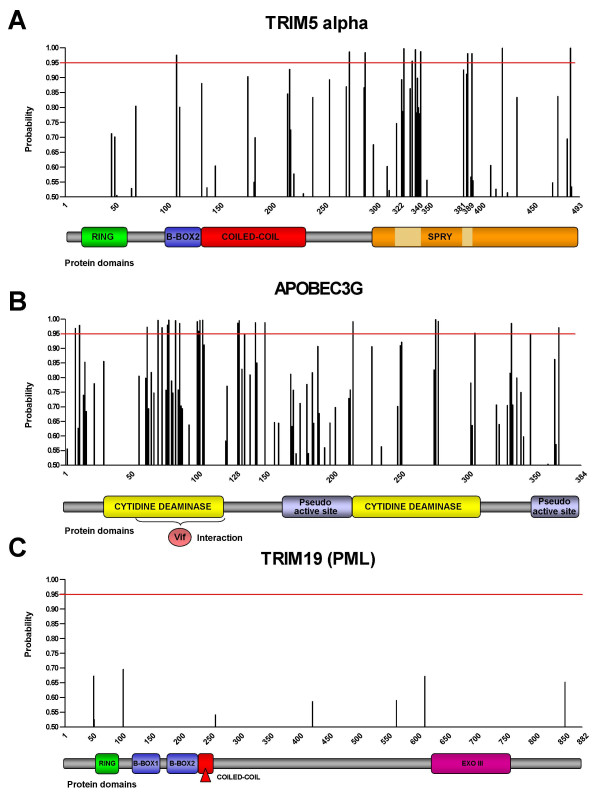
**Codons under positive selection in *TRIM5*α and *APOBEC3G***. Y-axis: Probabilities of positively selected codons (see text). X-axis: amino acid numbering and functional domains. *TRIM19 *is shown for comparison.

Using a recently developed Bayesian approach [16], we analyzed the site class under positive selection in *TRIM5*α and *APOBEC3G *in more detail. For *TRIM5*α, 11 of 493 (2%) codon sites can be predicted to be positively selected with high confidence (*P *> 0.95, Figure 2A). Two clusters of positive selection are found in the SPRY domain. The first cluster resides between amino acids 322 to 340 in the variable region 1 (v1, [18]), a region previously described as a "patch" of positive selection [3]. Replacement of the v1 region, or of specific amino acids within v1, modifies the restriction pattern of TRIM5α [19,20]. The second cluster, localized between amino acids 381 to 389, corresponds to the previously described variable region v2 of the SPRY domain [18]. Substitution of the human v2 region by a Rhesus monkey v2 exhibits no inhibitory activity against HIV-1 or a N-MLV_L117H_ chimera [19,20]. However, the role of v2 in species-specific lentiviral restriction has not yet been extensively tested.

The analysis also predicts a large number (24 of 384, 6%) of positively selected sites in the *APOBEC3G *(Figure 2B) sequence. This result is consistent with previous reports by Sawyer et al. [5]. However, the inclusion of several new species from an additional hominoid lineage, Hylobatidae (gibbons and siamangs), points to the existence of a cluster of residues under positive selection between amino acids 62 and 103, the region that defines the Vif-interaction domain [21]. The protein Vif, which counteracts the activity of APOBEC3G, is encoded by nearly all lentiviruses [22]. Within the Vif-interaction domain of APOBEC3G, 10 residues can be pinpointed to have evolved under strong positive selection. Interestingly, the APOBEC3G amino acid position 128, which controls the ability of the HIV-1 Vif protein to bind and inactivate this host defense factor [23,24], is correctly identified as being positively selected (*P *> 0.987).

The parallel assessment of multiple genes in the same set of primates allows for several considerations and conclusions. First, by including additional primate lineages, we modify and complement previously observed patterns for two antiviral defense genes/proteins. For TRIM5α, our analysis confirms previous results by Sawyer et al [3], but underscores the potential interest of the second variable region of the SPRY domain that may be of functional relevance and merits further experimental analysis. With respect to APOBEC3G, our analysis extends previous reports that showed protein-wide distribution of positively selected residues. It suggests that this protein potentially carries a functionally relevant cluster of selected residues that coincides with the region of HIV-1-Vif interaction [23,24]. Positive selected sites by Bayes Empirical Bayes Inference with probabilities P > 0.95 for the two proteins are listed in Additional file 3.

Second, the failure to identify signatures of positive selection in the *TRIM19 *(*PML*) gene suggests that its encoded protein does not have antiviral activity, or that the protein acts as an intermediary, lacking a physical protein-protein interaction with the pathogen. TRIM19 (PML) has been implicated in many functions, for example, in apoptosis and cell proliferation [9]. In addition, TRIM19 (PML) expression may act as an effector of the antiviral state induced by type I interferons [9]. Overexpression of TRIM19 (PML) is reported to confer resistance to infection by vesicular stomatitis virus and influenza A virus. Rabies, Lassa virus and lymphocytic choriomeningitis virus replicate to higher levels in PML-negative cells, whereas overexpression of the protein has no significant effect. Various roles have been proposed for TRIM19 (PML) in retroviral replication [8,25], although these findings remain controversial [26]. Many other viruses, including herpes simplex type 1 disturb the nuclear bodies that contain, among other proteins, TRIM19 (PML). However, it is unclear whether these effects are a consequence of the viral infection or a sign of its participation in antiviral defense. Thus, the effect of TRIM19 (PML) might be indirect. Failure to identify a signature of positive selection militates against a direct role of this protein in antiviral defense, because it would be expected that a prolonged contact with multiple pathogens over long evolutionary time periods would have resulted in signatures of positive selection indicative of a genetic conflict.

Finally, the absence of a signature of positive Darwinian selection in Cyclophilin A provides a complement to the understanding of the role of this protein in retroviral pathogenesis. Cyclophilin A interacts directly with the HIV-1 capsid, an interaction that may protect HIV-1 from antiviral restriction activity [27]. Although required by members of the HIV-1M/SIV_CPZ _lineage for replication, it is not needed by other primate immunodeficiency viruses [11]. Owl monkeys exhibit post-entry restriction of HIV-1 mediated by a TRIM5-Cyclophilin A fusion protein generated by retroposition [28]. Evolutionary analysis of *PPIA *indicates that Cyclophilin A has been preserved by strong purifying selection, leaving its protein sequence virtually unchanged. This is consistent with the interaction of Cyclophilin A and the viral capsid being limited to the HIV-1M/SIVcpz lineage.

Together, the results presented here further support that an evolutionary genomics approach may be very useful for systematically assessing functional roles of primate host proteins potentially relevant in viral pathogenesis [29]. Candidates for this approach may include other members of the TRIM or APOBEC families [30,31] as well as proteins involved in pathogen recognition and life cycle. Signatures of positive selection, but also the absence of signs of a genetic conflict, constitute relevant information for understanding the nature of virus-host protein interactions.

## Competing interests

The author(s) declare that they have no competing interests.

## Authors' contributions

MO carried out the molecular genetic studies, performed sequence and phylogenetic analysis and contributed to drafting of the manuscript. GB and RM carried out molecular genetic studies. HK conceived the study, performed the evolutionary genomic analyses and drafted the manuscript. AT conceived the study, supervised the molecular genetic analysis, assured funding, and drafted the manuscript.

## Supplementary Material

Additional file 1 GenBank accession numbers. Click here for file

Additional file 2 Primers for amplification and sequence analysis. Click here for file

Additional file 3 Positive selected sites by Bayes Empirical Bayes Inference with probabilities P > 0.95. Click here for file
