# Supplementary material for: Patterns of evolution of host proteins involved in retroviral pathogenesis
Source: Retrovirology. 2006 Feb 7;3:11. doi: 10.1186/1742-4690-3-11 (PMC1409793; doi:10.1186/1742-4690-3-11)
Supplement: Additional file 1 — GenBank accession numbers. [file 1742-4690-3-11-S1.doc]

Additional file 1. GenBank accession numbers.

| Name | Scientific name | Gene/Protein | **Accession Number** | **Reference** |
| --- | --- | --- | --- | --- |
| **Human** | Homo sapiens | TRIM5α | AY625000 | Yap,M.W., Proc. Natl. Acad. Sci. U.S.A. 101 (29), 10786-10791 (2004) |
|  |  | **TRIM19 (PML)** | NM_033238 | Turelli,P. Mol. Cell 7 (6), 1245-1254 (2001) |
|  |  | **APOBEC3G** | NM_021822 | Sheehy,A.M. Nature 418 (6898), 646-650 (2002) |
|  |  | **PPIA** | NM_021130 | Luban,J. Cell 73 (6), 1067-1078 (1993) |
| **Bonobo** | Pan paniscus | TRIM5α | DQ229282 | This work |
|  |  | **TRIM19 (PML)** | DQ231463 | This work |
|  |  | **APOBEC3G** | AH013832 | Sawyer,S.L. PLoS Biol. 2 (9), E275 (2004) |
|  |  | **PPIA** | DQ251275 | This work |
| **Chimpanzee** | Pan troglodytes | **TRIM5α** | AY923177 | This work |
|  |  | **TRIM19 (PML)** | DQ231464 | This work |
|  |  | **APOBEC3G** | AH013826 | Sawyer,S.L. PLoS Biol. 2 (9), E275 (2004) |
|  |  | **PPIA** | DQ251276 | This work |
| **Gorilla** | Gorilla gorilla | **TRIM5α** | AY923178 | This work |
|  |  | **TRIM19 (PML)** | DQ231465 | This work |
|  |  | **APOBEC3G** | AH013828 | Sawyer,S.L. PLoS Biol. 2 (9), E275 (2004) |
|  |  | **PPIA** | DQ251277 | This work |
| **Bornean orang-utan** | *Pongo pygmaeus* | **TRIM5α** | AY923179 | This work |
|  |  | **TRIM19 (PML)** | DQ231466 | This work |
|  |  | **APOBEC3G** | AH013829 | Sawyer,S.L. PLoS Biol. 2 (9), E275 (2004) |
|  |  | **PPIA** | DQ251278 | This work |
| **Lar gibbon** | *Hylobates lar* | **TRIM5α** | AY923180 | This work |
|  |  | **TRIM19 (PML)** | DQ231467 | This work |
|  |  | **APOBEC3G** | DQ251287 | This work |
|  |  | **PPIA** | DQ251279 | This work |
| **Nomascus** | *Hylobates leucogenys* | **TRIM5α** | DQ229283 | This work |
|  |  | **TRIM19 (PML)** | DQ231468 | This work |
|  |  | **APOBEC3G** | DQ251285 | This work |
|  |  | **PPIA** | DQ251280 | This work |
| **Siamang** | *Hylobates syndactylus* | **TRIM5α** | DQ229284 | This work |
|  |  | **TRIM19 (PML)** | DQ231469 | This work |
|  |  | **APOBEC3G** | DQ251286 | This work |
|  |  | **PPIA** | DQ251281 | This work |
| **Rhesus monkey** | *Macaca mulatta* | **TRIM5α** | AY625001 | Yap,M.W., Proc. Natl. Acad. Sci. U.S.A. 101 (29), 10786-10791 (2004) |
|  |  | **TRIM19 (PML)** | DQ231471 | This work |
|  |  | **APOBEC3G (1)** | AY331716 | Mariani,R. Cell 114 (1), 21-31 (2003) |
|  |  | **PPIA** | DQ251283 | This work |
| **African green monkey** | *Cercopithecus [Chlorocebus ] aethiops* | **TRIM5α** | AY669399 | Hatziioannou,T.P roc. Natl. Acad. Sci. U.S.A. 101 (29), 10774-10779 (2004) |
|  |  | **TRIM19 (PML)** | DQ231470 | This work |
|  |  | **APOBEC3G** | AY331714 | Mariani,R. Cell 114 (1), 21-31 (2003) |
|  |  | **PPIA** | DQ251282 | This work |
| **Cotton-top tamarin** | Saguinus oedipus | **TRIM5α** | DQ229285 | This work |
|  |  | **TRIM19 (PML)** | DQ231472 | This work |
|  |  | **APOBEC3G** | (2) | -- |
|  |  | **PPIA** | DQ251284 | This work |

(1) The NCBI sequence extended to include exon 1.

(2) Because of impossibility to complete the sequence of the Cotton-top tamarin, the sequence of the new world monkey,

red-chested mustached tamarin (*Saguinus labiatus*) APOBEC3G (AH013830) was used for analysis.
