# Supplementary material for: Patterns of evolution of host proteins involved in retroviral pathogenesis
Source: Retrovirology. 2006 Feb 7;3:11. doi: 10.1186/1742-4690-3-11 (PMC1409793; doi:10.1186/1742-4690-3-11)
Supplement: Additional file 2 — Primers for amplification and sequence analysis. [file 1742-4690-3-11-S2.doc]

Additional file 2. Primers for amplification and sequence analysis.

**Panel A-D.** Primer pairs. Exons were amplified by primers designed for the flanking regions. HotStarTaq Master Mix (QIAGEN) was used for PCR amplification of fragments smaller than 1 kb/ and Pfu polymerase (STRATAGENE) for fragments larger than 1 kb. [ ] PCR primer label. Underlined correspond to exonic primers all other primers are placed in introns. Both strands from PCR products were sequenced. *PCR performed on cDNA. **Panel E.** Primer sequences

## A

| **APOBEC3G** | **Exon 1** | **Exon 2** | **Exon 3** | **Exon 4** | **Exon5** | **Exon 6** | **Exon 7** | **Exon 8** |
| --- | --- | --- | --- | --- | --- | --- | --- | --- |
| Gibbon | Failed | Failed | [SG394/SG395]  [SG394/SG1328] | [SG394/SG1328] | [SG1329/SG1330] | [SG414/SG415] | [SG416/SG421] | [SG416/SG421] |
| **Nomascus** | [SG388/SG389] | [SG390/SG391] | [SG394/SG395]  [SG394/SG1328] | [SG394/SG1328] | [SG412/SG413]  [SG412/SG1330] | [SG414/SG415] | [SG416/SG419] | [SG418/SG421] |
| **Siamang** | [SG388/SG389] | [SG390/SG391] | [SG394/SG395] | [SG394/SG1328] | [SG412/SG413]  [SG412/SG1330] | [SG414/SG415] | [SG416/SG421] | [SG418/SG421] |
| **Rhesus Monkey** | [SG388/SG389] | Available from NCBI | Available from NCBI | Available from NCBI | Available from NCBI | Available from NCBI | Available from NCBI | Available from NCBI |

#### B

| **PPIA** | **Exon 1** | **Exon 2** | **Exon 3** | **Exon 4** | **Exon5** |
| --- | --- | --- | --- | --- | --- |
| Bonobo | [SG1242/SG63] | [SG54/SG65] | [SG54/SG65] | [SG54/SG65]  [SG56/SG65] | [SG58/SG61] |
| **Chimpanzee** | [SG1242/SG63] | [SG54/SG65] | [SG54/SG65] | [SG54/SG65]  [SG56/SG65] | [SG58/SG61] |
| **Gorilla** | [SG1242/SG63] | [SG54/SG65] | [SG54/SG65] | [SG54/SG65]  [SG56/SG65] | [SG58/SG61] |
| **Bornean orang-utan** | [SG1242/SG63] | [SG54/SG1247] | [SG54/SG1247] | [SG56/SG65] | [SG58/SG61] |
| **Lar gibbon** | [SG1305/SG63] | [SG54/SG65] | [SG54/SG65] | [SG54/SG65]  [SG56/SG65] | [SG58/SG59] |
| **Nomascus** | [SG1305/SG63] | [SG54/SG65] | [SG54/SG65] | [SG56/SG65] | [SG58/SG59] |
| **Siamang** | [SG1305/SG63] | [SG54/SG65] | [SG54/SG65] | [SG54/SG65]  [SG56/SG65] | [SG58/SG59] |
| **Rhesus monkey** | [SG1242/SG1247]* | [SG1242/SG1247]*  [SG1244/SG1247]*  [SG54/SG1247] | [SG1242/SG1247]*  [SG1244/SG1247]*  [SG1245/SG1243]*  [SG54/SG1247] | [SG1245/SG1243]*  [SG56/SG65] | [SG1245/SG1243]* |
| **African green monkey** | [SG1242/SG63] | [SG54/SG65] | [SG54/SG65] | [SG54/SG65]  [SG56/SG65] | [SG58/SG61] |
| **Cotton-top tamarin** | [SG1242/SG1243]* | [SG1242/SG1243]*  [SG54/SG1247] | [SG1242/SG1243]*  [SG54/SG1247] | [SG1242/SG1243]* | [SG1242/SG1243]* |

C

| ***TRIM19 (PML)*** | **Exon 1** | **Exon 2** | **Exon 3** | **Exon 4** | **Exon5** | Exon6 | **Exon7** | **Exon8** | Exon9 |
| --- | --- | --- | --- | --- | --- | --- | --- | --- | --- |
| Bonobo | [SG331/SG332] | [SG333/SG334]  [SG335/SG336] | [SG337/SG520]  [SG521/SG340] | [SG343/SG344] | [SG345/SG346] | [SG347/SG348] | [SG349/SG350] | [SG1205/SG368] | [SG371/SG372]  [SG373/SG374]  [SG375/SG1178] |
| **Chimpanzee** | [SG331/SG332] | [SG333/SG334]  [SG335/SG336] | [SG337/SG520]  [SG521/SG340] | [SG343/SG344] | [SG345/SG346] | [SG347/SG348] | [SG349/SG350] | [SG1205/SG368] | [SG371/SG372]  [SG373/SG374]  [SG375/SG1178] |
| **Gorilla** | [SG331/SG332] | [SG333/SG334]  [SG335/SG336] | [SG337/SG520]  [SG521/SG340] | [SG343/SG344] | [SG345/SG346] | [SG347/SG348] | [SG349/SG350] | [SG367/SG370] | [SG371/SG372]  [SG373/SG374]  [SG375/SG1178] |
| **Bornean orang-utan** | [SG331/SG332] | [SG333/SG334]  [SG335/SG336] | [SG337/SG520]  [SG521/SG340] | [SG343/SG344] | [SG345/SG346] | [SG347/SG348] | [SG349/SG350] | [SG367/SG368] | [SG371/SG372]  [SG373/SG374]  [SG375/SG1178] |
| **Lar gibbon** | [SG331/SG332] | [SG333/SG334]  [SG335/SG336] | [SG337/SG520]  [SG521/SG340] | [SG343/SG344] | [SG345/SG346] | [SG347/SG348] | [SG349/SG350] | [SG1205/SG368] | [SG371/SG372]  [SG373/SG374]  [SG375/SG1178] |
| **Nomascus** | [SG331/SG332] | [SG333/SG334]  [SG335/SG336] | [SG337/SG520]  [SG521/SG340] | [SG343/SG344] | [SG345/SG346] | [SG347/SG348] | [SG349/SG350] | [SG367/SG368] | [SG371/SG372]  [SG373/SG374]  [SG375/SG1178] |
| **Siamang** | [SG331/SG332] | [SG333/SG334]  [SG335/SG336] | [SG337/SG520]  [SG521/SG340] | [SG343/SG344] | [SG345/SG346] | [SG347/SG348] | [SG349/SG350] | [SG367/SG368] | [SG371/SG372]  [SG373/SG374]  [SG375/SG1178] |
| **Rhesus monkey** | [SG1250/SG1128]  [SG1227/SG1228] | [SG333/SG334]  [SG335/SG336] | [SG337/SG520]  [SG521/SG340] | [SG343/SG344] | [SG345/SG346] | [SG347/SG350]  [SG1183/SG1184]* | [SG347/SG350]  [SG1183/SG1184]* | [SG367/SG368] | [SG371/SG372]  [SG373/SG374]  [SG375/SG1178] |
| **African green monkey** | [SG331/SG332] | [SG333/SG334]  [SG335/SG336] | [SG337/SG520]  [SG521/SG340] | [SG343/SG344] | [SG345/SG346] | [SG1183/SG1184]* | [SG1183/SG1184]* | [SG1185/SG372]* | [SG371/SG372]  [SG373/SG1178]  [SG371/SG1178]  [SG375/SG1178] |
| **Cotton-top tamarin** | [SG1275/SG332] | [SG333/SG334]  [SG1212/SG1213]* | [SG1212/SG1213]* | [SG522/SG344] | [SG1183/SG1184]* | [SG1183/SG1184]* | [SG1183/SG1184]*  [SG1217/SG1218]* | [SG367/SG368] | [SG371/SG372] [SG373/SG374]  [SG373/SG1211] |

D

| **TRIM5α** | **Exon2** | **Exon4** | Exon5 | **Exon6** |
| --- | --- | --- | --- | --- |
| Bonobo | [SG883/SG884]  [SG885/SG886]  [SG897/SG898] | [SG949/SG950] | [SG964/SG965] | [SG966/SG967]  [SG968/SG969]  [SG970/SG971]  [SG972/SG973] |
| **Chimpanzee** | [SG883/SG884]  [SG885/SG886]  [SG897/SG898] | [SG949/SG950] | [SG964/SG965] | [SG966/SG967]  [SG968/SG969]  [SG970/SG971]  [SG972/SG973] |
| **Gorilla** | [SG883/SG884]  [SG885/SG886]  [SG897/SG898] | [SG949/SG950] | [SG964/SG965] | [SG966/SG967]  [SG968/SG969]  [SG970/SG971]  [SG972/SG973] |
| **Bornean orang-utan** | [SG883/SG884]  [SG885/SG886]  [SG897/SG898] | [SG949/SG950] | [SG964/SG965] | [SG966/SG967]  [SG968/SG969]  [SG970/SG971]  [SG972/SG973] |
| **Lar gibbon** | [SG883/SG884]  [SG885/SG886]  [SG897/SG898] | [SG949/SG950] | [SG964/SG965] | [SG966/SG967]  [SG968/SG969]  [SG970/SG971]  [SG970/SG975] |
| **Nomascus** | [SG883/SG884]  [SG885/SG886]  [SG897/SG898] | [SG949/SG950] | [SG964/SG965] | [SG966/SG967]  [SG968/SG969]  [SG970/SG971]  [SG972/SG973] |
| **Siamang** | [SG883/SG884]  [SG885/SG886]  [SG897/SG898] | [SG949/SG950] | [SG964/SG965] | [SG966/SG967]  [SG968/SG969]  [SG970/SG971]  [SG972/SG973] |
| **Cotton-top tamarin** | [SG883/SG888]  [SG1156/SG1157]*  [SG1153/SG1150]* | [SG1151/SG1150]*  [SG1153/SG1150]* | [SG1151/SG1150]*  [SG1153/SG1150]* | [SG970/SG977]  [SG1153/SG1150]*  [SG1151/SG1150]* |

**E**

**Primer code** Sequence (5’→ 3’)

| **SG** | **54** | CTAAAGACATGGGTACTAAGCAAC |
| --- | --- | --- |
| **SG** | **56** | AGTTTGAACCTTGCAGATTTGGC |
| **SG** | **58** | AGCTACCTTTCTCGTCTTGGTTC |
| **SG** | **59** | GAGAGCACAAAGATTCTAGGATAC |
| **SG** | **61** | GTTTCAGAAGTAACCCATTACTGC |
| **SG** | **63** | GCCCCATTCCCGCACGCCG |
| **SG** | **65** | AGGTGGTTAGTGTGCCATGTTGT |
| **SG** | **331** | CTTCACGCACTCCAAGATCT |
| **SG** | **332** | CCACCACAGCAAACCAAC |
| **SG** | **333** | GACTTCTCCAGGCCTCAC |
| **SG** | **334** | CACACAGCCTGCGCATCCAC |
| **SG** | **335** | TCGAGAGTCTGCAGCGGCG |
| **SG** | **336** | TCTACCTGGTACTTGGATGC |
| **SG** | **337** | CATGCCATGATTCAAAGTCTGG |
| **SG** | **340** | CGTAGCACTTCATCCTCTGCA |
| **SG** | **343** | CATGTCCTTGACCTGCCTG |
| **SG** | **344** | CACAACCTGCCTGACCTGCT |
| **SG** | **345** | GCTGCTGCCTAGTCATTTCT |
| **SG** | **346** | CTGGTGGAACTCAGGCCTTC |
| **SG** | **347** | ACCTGACCTGGCTCTGTGACTC |
| **SG** | **348** | AGCACCCAGGCCACTGC |
| **SG** | **349** | ATGCATCCTAGGCAGTTCA |
| **SG** | **350** | TCCAGGCATGCAGCTGTCATTTG |
| **SG** | **367** | CCCTCTGAATCCCTGACG |
| **SG** | **368** | AGTTGTTGGCCAGAGCTCAG |
| **SG** | **370** | GTGCACTTGTGGAGGCAT |
| **SG** | **371** | CAGAGCTCTCTGTGACCCTGGGTC |
| **SG** | **372** | CGGATGAGAGGCAGGGCAGC |
| **SG** | **373** | CAAACTTCTTCCGGGCCCT |
| **SG** | **374** | CAGCACAGCTGCCTGCACCAG |
| **SG** | **375** | CAGCTGGCCCAGCATGTCTA |
| **SG** | **388** | AATTGCCTTGGGTCCTGCCG |
| **SG** | **389** | GGCCCAGTGGGTAGAGCG |
| **SG** | **390** | CCCAGGAGTGCTCTCTACATT |
| **SG** | **391** | TTAGCTCCTGCCTGCAAAG |
| **SG** | **394** | AAGATCTCCTGGACTCCTGTC |
| **SG** | **395** | CAGAAGTAGTAGAGGCGG |
| **SG** | **412** | CAGGAGGGCTCTTACTCCTC |
| **SG** | **413** | TACCACCCAGAACCTTCT |
| **SG** | **414** | GATTACCTCATGGCTTGCTTTC |
| **SG** | **415** | CATCCTACTCCAGGCTTCCATTC |
| **SG** | **416** | CACAGCGGGAGTGTGACTTA |
| **SG** | **418** | ACTCGCTCACCCTTTGCTC |
| **SG** | **419** | GATTCAAAGTAATGCACTCTA |
| **SG** | **421** | GACATCTACACATTTGCTAGTG |
| **SG** | **520** | CAAAGGCACTATCCTGCTCCT |
| **SG** | **521** | CAGAGATCCAGCAGCGACAG |
| **SG** | **522** | CGACTACGAGGAGATGGCCA |
| **SG** | **883** | TCCCATTTTAACCTTCCCAATC |
| **SG** | **884** | GGCGTGCACAATGATCAAC |
| **SG** | **885** | GATCAGTTACCAGCCTGAGAACATA |
| **SG** | **886** | CAGGTCTATCATGACAAGGCAGT |
| **SG** | **897** | AGCCTCTGCCTGGTAGACTGAGT |
| **SG** | **898** | CACAGCAACGCAGAGAAGGAA |
| **SG** | **949** | CCATTCTCAGCACATTAGGAACC |
| **SG** | **950** | TTCTGCCCTGGGAGATGTTT |
| **SG** | **964** | AGAGAGTTCAAGTGCCTCAATGG |
| **SG** | **965** | AACACCACTTGAATTCCATAACCC |
| **SG** | **966** | CAGTGCTGACTCCTTTGTTTGTATTC |
| **SG** | **967** | GAGCCACTGTCACATCAACTGTAGA |
| **SG** | **968** | CAAGGTTCCTCCCAGTTTTCTCTC |
| **SG** | **969** | GGTTGGAAGCCAGCACATACC |
| **SG** | **970** | GCTCTCAAAGTATCACATCAGGGA |
| **SG** | **971** | GAGAAATCCATGGTTTGTGATATTGA |
| **SG** | **972** | CTAGACTATGAGGCTTGCACTGTCT |
| **SG** | **973** | ACAATATGGCACAAGGCAATTATTA |
| **SG** | **975** | GAGAGACAGGAGTTGAACTGAGATCC |
| **SG** | **977** | TCCCAGCTACTTGGGAGGCT |
| **SG** | **1050** | CAAGGGCACGAGCTATG |
| **SG** | **1051** | GGTGTCACAGGAAGAGAT |
| **SG** | **1053** | GGTACTGATCATTGGCTCGAA |
| **SG** | **1056** | TACGCGGACTACCAAGATCAAG |
| **SG** | **1057** | CTGCAGGGGGTGCCAACACTC |
| **SG** | **1178** | TCTCTGCCCACCAGAGACTCC |
| **SG** | **1183** | GAGGCAGAGAGAGTGAAGGCC |
| **SG** | **1184** | CTGAGTCTTCCGAGCTGCTGA |
| **SG** | **1185** | CCAGAAGAGGAAGTGCAGCCAG |
| **SG** | **1205** | GGAGCAGACATCTCAGGTCCTG |
| **SG** | **1211** | TGGAACCTTCTCTGTAGGATGTTTAGAG |
| **SG** | **1212** | AAGCCAAGTGCCCGAAGCT |
| **SG** | **1213** | GGATGCGCTGTAGCACAGC |
| **SG** | **1217** | CTAGCACCTCCAAGGCAGTCTC |
| **SG** | **1218** | TCATTGTCAAGCTTGAGGTCAAAG |
| **SG** | **1227** | CTTTACCGTAAGTCAGCGGTAGG |
| **SG** | **1228** | CACTGACCCTTCTCACCTTGC |
| **SG** | **1242** | CTCGTGCCGTTTTGCAGAC |
| **SG** | **1243** | CCTGAGCTACAGAAGGAATGATCTG |
| **SG** | **1244** | CTTCGACATTGCCGTCGAC |
| **SG** | **1245** | CAGACAAGGTCCCAAAGACAGC |
| **SG** | **1247** | CCATGGACAAGATGCCAGGA |
| **SG** | **1250** | CTTCCCATCACTCACAGAACTG |
| **SG** | **1275** | TAGACCATCGAAACTAAGCTGG |
| **SG** | **1305** | GGCCGAACGTGGTATAAAAGG |
| **SG** | **1328** | GATGAGCAGGAGGTGAGAGGA |
| **SG** | **1329** | TCTGACAGGTGCTAAGGGATGTG |
| **SG** | **1330** | AATCCTGGAGAGTCACTGAAGCC |
