# Supplementary material for: Patterns of evolution of host proteins involved in retroviral pathogenesis
Source: Retrovirology. 2006 Feb 7;3:11. doi: 10.1186/1742-4690-3-11 (PMC1409793; doi:10.1186/1742-4690-3-11)
Supplement: Additional file 3 — Positive selected sites by Bayes Empirical Bayes Inference with probabilities P > 0.95. [file 1742-4690-3-11-S3.doc]

**Additional file 3.** Positive selected sites by Bayes Empirical Bayes Inference with probabilities P>0.95. Shown is human amino acid numbering of TRIM5α and APOBEC3G as reference.

| Position | Amino acid | Probability |
| --- | --- | --- |
| **TRIM5α** | | |
| 107 | Q | 0.976 |
| 272 | Q | 0.987 |
| 287 | V | 0.984 |
| 324 | K | 0.998 |
| 332 | R | 0.956 |
| 335 | R | 0.994 |
| 340 | V | 0.988 |
| 385 | C | 0.981 |
| 389 | K | 0.981 |
| 418 | F | 0.999 |
| 483 | G | 0.999 |
| APOBEC3G | | |
| 8 | T | 0.969 |
| 11 | R | 0.979 |
| 61 | E | 0.973 |
| 69 | R | 0.996 |
| 72 | H | 0.971 |
| 76 | K | 0.980 |
| 77 | W | 0.997 |
| 82 | R | 0.995 |
| 85 | E | 0.986 |
| 98 | T | 0.992 |
| 99 | K | 0.959 |
| 101 | T | 0.996 |
| 102 | R | 0.997 |
| 128 | D | 0.987 |
| 129 | P | 0.995 |
| 141 | K | 0.989 |
| 148 | T | 0.989 |
| 213 | R | 0.992 |
| 274 | D | 0.999 |
| 276 | D | 0.993 |
| 303 | K | 0.953 |
| 330 | E | 0.986 |
| 344 | K | 0.951 |
| 365 | D | 0.971 |
